# Pediatric-type high-grade gliomas with *PDGFRA* amplification in adult patients with Li-Fraumeni syndrome: clinical and molecular characterization of three cases

**DOI:** 10.1186/s40478-024-01762-7

**Published:** 2024-04-11

**Authors:** Yuji Kibe, Fumiharu Ohka, Kosuke Aoki, Junya Yamaguchi, Kazuya Motomura, Eiji Ito, Kazuhito Takeuchi, Yuichi Nagata, Satoshi Ito, Nobuhiko Mizutani, Yoshiki Shiba, Sachi Maeda, Tomohide Nishikawa, Hiroki Shimizu, Ryuta Saito

**Affiliations:** 1 Department of Neurosurgery, Nagoya University Graduate School of Medicine, 65 Tsurumai-cho, Showa-ku, Nagoya, 466-8550 Japan; 2 Department of Neurosurgery, Konan Kosei Hospital, 137 Oomatsubara, Takaya-cho, Konan, 483-8703 Japan

**Keywords:** Li-Fraumeni syndrome, Pediatric-type high-grade glioma, H3-wildtype and IDH-wildtype, *PDGFRA* amplification

## Abstract

Li-Fraumeni syndrome (LFS) is an autosomal dominant tumor predisposition syndrome caused by heterozygous germline mutations or deletions in the *TP53* tumor suppressor gene. Central nervous system tumors, such as choroid plexus tumors, medulloblastomas, and diffuse gliomas, are frequently found in patients with LFS. Although molecular profiles of diffuse gliomas that develop in pediatric patients with LFS have been elucidated, those in adults are limited. Recently, diffuse gliomas have been divided into pediatric- and adult-type gliomas, based on their distinct molecular profiles. In the present study, we investigated the molecular profiles of high-grade gliomas in three adults with LFS. These tumors revealed characteristic histopathological findings of high-grade glioma or glioblastoma and harbored wild-type *IDH1/2* according to whole exome sequencing (WES). However, these tumors did not exhibit the key molecular alterations of glioblastoma, IDH-wildtype such as *TERT* promoter mutation, *EGFR* amplification, or chromosome 7 gain and 10 loss. Although WES revealed no other characteristic gene mutations or copy number alterations in high-grade gliomas, such as those in histone H3 genes, *PDGFRA* amplification was found in all three cases together with uniparental disomy of chromosome 17p, where the *TP53* gene is located. DNA methylation analyses revealed that all tumors exhibited DNA methylation profiles similar to those of pediatric-type high-grade glioma H3-wildtype and IDH-wildtype (pHGG H3-/IDH-wt), RTK1 subtype. These data suggest that high-grade gliomas developed in adult patients with LFS may be involved in pHGG H3-/IDH-wt. *PDGFRA* and homozygous alterations in *TP53* may play pivotal roles in the development of this type of glioma in adult patients with LFS.

**Supplementary Information:**

The online version contains supplementary material available at 10.1186/s40478-024-01762-7.

## Introduction

Li-Fraumeni syndrome (LFS) is a rare autosomal dominant tumor predisposition syndrome caused by heterozygous germline mutations or deletions in the *TP53* tumor suppressor gene on chromosome 17p13. The Chompret criteria, which include items on family history, multiple cancers, rare cancers, and juvenile breast cancer, are widely used to perform *TP53* genetic testing for suspected LFS. The latest update on the description of Li-Fraumeni syndrome and its clinical presentation was revised in 2015 [1]. Brain tumors develop in 16.4% of patients with LFS, leading to LFS core cancer [2]. The histological features of LFS-associated brain tumors are unevenly distributed among patients of different ages, with a predominance of choroid plexus tumors in infants, followed by medulloblastomas in children and mostly diffuse gliomas in adults. The peak age of glioma onset in adults occurs slightly earlier than that of adult brain tumors with sporadic *TP53* mutations [3, 4].

The mechanism of tumorigenesis in cancer predisposition syndrome is suggested to differ from that in sporadic cancer. Germline mutations in cancer predisposition genes play a pivotal role in pediatric cancer development. Pediatric cancers have been reported to have genomic characteristics that are distinct from those of adult cancers, such as lower mutation rates and frequent single driver mutations [5, 6]. These data suggest that diffuse gliomas developing in the setting of LFS, even in adult cases, may reveal distinct clinical characteristics and molecular profiles from those of most adult-type gliomas. However, previous reports about the clinical course and molecular alterations in this type of glioma especially in adults are limited [7–9].

According to the World Health Organization (WHO), 5th edition of the classification of central nervous system tumors (WHO CNS 5), diffuse gliomas are classified into adult and pediatric types. Adult-type gliomas are divided into isocitrate dehydrogenase (IDH)-wildtype and IDH-mutant type. Glioblastoma, IDH-wildtype is molecularly characterized by *TERT* promoter (*TERT*p) mutation, *EGFR* amplification, or chromosome 7 gain and 10 loss (7 +/10−), in addition to characteristic pathological findings such as necrosis and microvascular proliferation. Pediatric-type diffuse gliomas have molecular features distinct from adult-type gliomas and lack *IDH* mutations, one of the representative gene alterations in adult-type gliomas, although the two types share an overlapping histology [10]. The most important genetic alterations in pediatric-type high-grade gliomas are those of histone H3 genes. Pediatric high-grade gliomas exhibiting histone H3-K27 or -G34 alterations are subdivided into diffuse midline glioma, H3 K27-altered or diffuse hemispheric glioma, H3 G34-mutant. DNA methylation profiling revealed that a subset of other pediatric-type high-grade gliomas without *IDH* and histone H3 mutations, defined as diffuse pediatric-type high-grade gliomas, H3-wildtype and IDH-wildtype (pHGG H3-/IDH-wt), also have distinct molecular profiles from adult-type glioblastoma, IDH-wildtype [11]. The molecular mechanisms underlying the development of this pediatric-type glioma has not yet been clarified.

Here, we investigated three cases of malignant gliomas that developed in adult patients with LFS. Although the tumors in our cases possessed typical histopathological features of high-grade glioma or glioblastoma and did not harbor *IDH* mutations, their molecular features were distinct from those of typical glioblastoma, IDH-wildtype. Further analysis revealed *PDGFRA* amplification and uniparental disomy (UPD) of the *TP53* locus in all cases. All three tumors revealed DNA methylation profiles similar to those of pHGG H3-/IDH-wt.

## Materials and methods

### Etiology

This study was approved by the Institutional Review Board of Nagoya University Hospital (approval number: 2021-0451) and complied with all provisions of the World Medical Association Declaration of Helsinki.

### Patient data

We encountered three cases of high-grade gliomas in adult patients with LFS between January 2020 and February 2021 at Nagoya University Hospital (Nagoya, Japan). Patient data on clinical information and outcomes, including age, sex, past medical history, family history, histopathological findings, extent of resection, prescribed adjuvant therapy, radiographic findings before and after treatment, progression-free survival (PFS), and overall survival (OS) were retrospectively analyzed. PFS and OS were defined as the duration from initial surgery to recurrence and the duration from initial surgery to death, respectively.

### DNA extraction from tumor and blood samples

Tumor samples were obtained intraoperatively. DNA was extracted from frozen tumors and blood samples using a QIAamp DNA Mini Kit (Qiagen, Hilden, Germany) according to the manufacturer’s instructions. For Case 2, we extracted DNA from formalin-fixed paraffin-embedded (FFPE) samples of recurrent tumors using the GeneRead DNA FFPE Kit (Qiagen) according to the manufacturer’s instructions. The amount of DNA obtained was evaluated using a Qubit dsDNA HS Assay Kit (Invitrogen, Paisley, Scotland).

### Whole exome sequencing

Whole exome sequencing (WES) was performed using targeted capture of all exon sequences with a SureSelect Human All Exon Kit v6 (Agilent Technologies), followed by sequencing on the NovaSeq 6000 platform (Illumina, San Diego, CA, USA) in 150-bp paired-end mode or on the MiSeq platform (Illumina) in 75-bp paired-end mode. A median of 69,308,761 reads per sample was obtained and aligned to cover the hg19 reference genome with 174 × coverage using the Burrows-Wheeler aligner (http://bio-bwa.sourceforge.net/) with default parameters and the -mem option. The Mutect2 tumor-only mode in the Genome Analysis ToolKit (GATK) was used for variant calling. The identified mutations were annotated using the ANNOVAR software. We also evaluated the mismatch ratio of common single nucleotide polymorphisms (SNPs), which have been reported at a frequency of > 5%. The B allele frequency (BAF) was calculated using the mismatch ratio and (1—the mismatch ratio) in each SNP region. Higher or lower values of the mismatch ratio and (1—the mismatch ratio) are indicated by yellow or blue dots, respectively (Fig. 4B–D).

### Genome-wide DNA methylation analysis

The Illumina Infinium Human MethylationEPIC (EPIC) BeadChip array (Illumina, San Diego, CA, USA) was used for genome-wide methylation analysis. In total, 500 ng of DNA extracted from frozen specimens or FFPE samples was used as the input material. The output data (IDAT files) were checked for general quality as indicated by the manufacturer. Preprocessing for the analysis of the output data and calculation of the beta score was performed with the Minfi package using R software, version 3.4.1 [12]. After filtering using the ChAMP package, the remaining probes for analysis totaled 384,629 [13]. The beta scores were normalized using the BMIQ method in the ChAMP package. The top 10,000 highest median absolute deviation (MAD) probes on CpG islands were selected for analysis. From the beta scores of the top 10,000 MAD probes, a distance matrix was generated using the Ward method and visualized by t-distributed stochastic neighbor embedding (t-SNE) in two dimensions using the Rtsne package. Reference methylation data for glioblastoma, IDH-wildtype (GSE109381) and pediatric-type glioma (GSE131482) were obtained from the Gene Expression Omnibus database (http://www.ncbi.nlm.nih.gov/geo/) for comparison. A molecular classification algorithm and copy number analysis from the German Cancer Center (DKFZ classifier, https://www.molecularneuropathology.org/mnp/) [14] was performed.

### Copy number variant analysis

Copy number variant (CNV) analysis was performed according to the GATK Best Practice using WES data, as described in our previous study [15]. Gene-level focal CNVs were identified by intersecting the gene boundaries with segment intervals and calculating the weighted log2 copy ratio of the gene. Copy number plots were also generated using the methylation classifier data. The same set of data generated by employing the Illumina 450 k or Illumina 850 k/EPIC arrays can be used to calculate CNVs using the ‘conumee’ package for R (http://bioconductor.org/packages/conumee). A log2 ratio ± 0.35 was used as the cutoff for amplification/loss and a log2 ratio − 0.415 was used as the cut-off for homozygous loss. To identify significantly recurrent copy number amplifications and deletions at arm level and focal level, focal CNV was defined as affected regions spanning less than 50% of a chromosome arm [16].

## Results

### Clinical characteristics of the patients

**Fig. 1:**
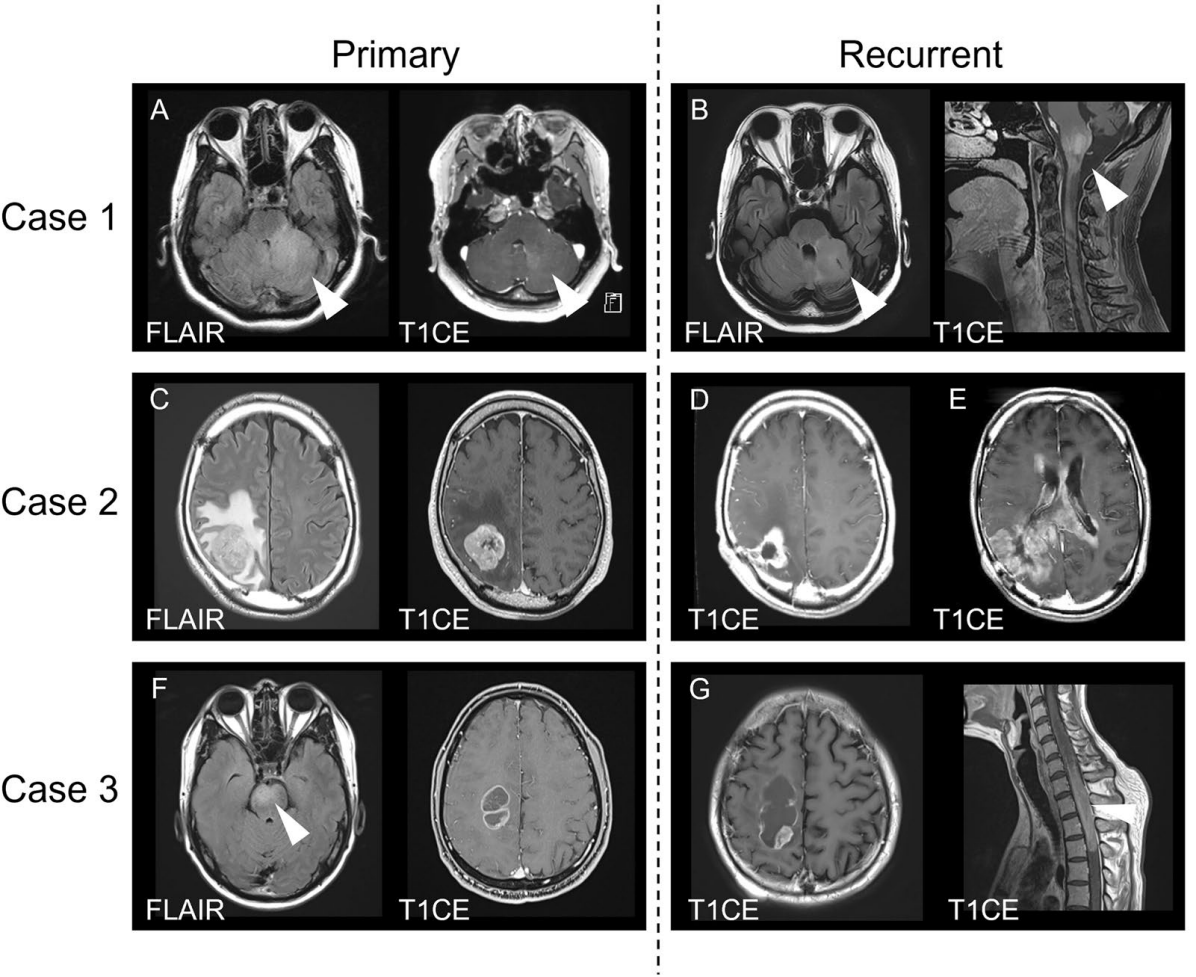
MRI of primary and recurrent tumors in the three cases. **A** FLAIR (left) and contrast-enhanced T1-weighted (right) images showing the primary tumor in Case 1. **B** FLAIR (left) and contrast-enhanced T1-weighted (right) images showing the recurrent tumor in Case 1. **C** FLAIR (left) and contrast-enhanced T1-weighted (right) images showing the primary tumor in Case 2. **D** Contrast-enhanced T1-weighted image showing the first recurrent tumor in Case 2. **E** Contrast-enhanced T1-weighted image showing the second recurrent tumor in Case 2. **F** FLAIR (left) and contrast-enhanced T1-weighted (right) images showing the primary tumor in Case 3. **G** Contrast-enhanced T1-weighted images showing a recurrent intracranial tumor (left) and cervical tumor (right) in Case 3. T1CE: contrast-enhanced T1-weighted imaging

The clinical characteristics of the patients are summarized in Table 1. Three patients with LFS developed malignant gliomas between January 2020 and February 2021 at Nagoya University. All patients met Chompret’s criteria for LFS [1] based on their past medical and family histories. The mean age at diagnosis was 40 years (range: 32–45). Two patients were male. Two patients had a family history of brain tumors. None of the patients had a history of previous radiation exposure. Magnetic resonance imaging (MRI) revealed a left cerebellar peduncle lesion with faint contrast enhancement (Case 1), a right parietal lobe lesion with nodular contrast enhancement (Case 2), a right parietal lobe lesion with ring enhancement, and a lesion in the pons (no enhancement; Case 3) (Fig. 1). Histopathological findings of the collected lesions were consistent with those of high-grade glioma (Case 1) and glioblastoma (Cases 2 and 3). All patients underwent postoperative chemoradiotherapy. The mean progression free survival was 10.4 months (range: 5.1–17.0). All patients died 18.5 months (range: 15.3–21.7) after the initial surgery.

**Table 1:** Summary of clinical characteristics and clinical course of three cases

	Case 1	Case 2	Case 3
Sex	Female	Male	Male
Age	43	45	32
Past medical history	Pituitary adenoma (36y)	Mandibular osteosarcoma (26y)	None
	Left adrenal adenoma (36y)	Gastric cancer (42y)	
	Bilateral breast cancer (40y)	Colon cancer (44y)	
Family history	Mother; breast cancer (38y)	Grand father; gastric cancer (70y)	Mother; bilateral breast cancer (32y) Brother; brain tumor (17y)
	Daughter; osteosarcoma (18y)	Grand mother; gastric cancer (50y)	
	Son; medulloblastoma (10y)	Aunt; pancreatic cancer (68y)	
Symptom	Left hemiparesis	Gait disturbance	Left hemiparesis
	Dysarthria		Left sensory disturbance
Tumor location	Left cerebellar peduncle	Right parietal lobe	Right pons
			Right parietal lobe
Surgery	Biopsy	Subtotal resection	Partial resection
Adjuvant therapy	TMZ, BEV	TMZ	TMZ, TTF
	Focal irradiation (60 Gy/30fr)	Focal irradiation (60 Gy/30fr)	Focal irradiation (60 Gy/30fr)
Salvage therapy	Best supportive care	Gross total removal	BEV
		TMZ, BEV, TTF	Focal irradiation (20 Gy/5Fr)
PFS (months)	17.0	5.1	9.2
OS (months)	21.7	18.6	15.3

### Case presentations

#### Case 1

A 43-year-old female presented with left hemiparesis and dysarthria. The patient had past medical history of pituitary adenoma (age 36 years), left adrenal adenoma (age 36) and bilateral breast cancer (age 40). The patient also had family histories of breast cancer (mother), osteosarcoma (daughter), and medulloblastoma (son). MRI revealed a left cerebellar peduncle lesion with faint contrast enhancement (Fig. 1A). Histopathological findings of the tumor specimen obtained by biopsy were consistent with those of a high-grade glioma. The patient underwent extended focal irradiation of 60 Gy/30 Fr with concomitant and adjuvant temozolomide (TMZ)　(Stupp protocol [17]) together with bevacizumab (BEV). However, the tumor infiltrated the medulla oblongata 17.0 months after the surgery (Fig. 1B). The patient died 21.7 months after the biopsy.

#### Case 2

A 45-year-old male presented with gait disturbance. The patient had past medical history of mandibular osteosarcoma (age 26 years), gastric cancer (age 42) and colon cancer (age 44). The patient also had family histories of gastric cancer (grandfather and grandmother) and pancreatic cancer (aunt). MRI revealed a lesion with nodular contrast enhancement in the right parietal lobe (Fig. 1C). Subtotal resection was performed. The histopathological findings were consistent with those of glioblastoma. The patient underwent Stupp protocol. Only 5.1 months later, a recurrent tumor appeared in the surgical cavity (Fig. 1D). Subsequently, a second tumor resection was performed. Gross total resection was achieved. Despite multidisciplinary treatment including TMZ, BEV, and tumor-treating fields (TTFs), a second recurrent tumor infiltrating the left hemisphere through the corpus collosum appeared 11.1 months after the second surgery (Fig. 1E). The patient died 18.6 months after the first surgery.

#### Case 3

A 32-year-old male presented with left-sided hemiparesis and dysesthesia. Although the patient had no relevant medical history, he had a family history of bilateral breast cancer (mother) and a brain tumor (brother). MRI revealed lesions in the pons (no enhancement) and right parietal lobe (ring enhancement) (Fig. 1F). A partial resection of the right parietal lesion was performed. The histopathological findings were consistent with those of glioblastoma. The patient underwent Stupp protocol. TTF treatment was also introduced after the completion of irradiation with maintenance therapy for TMZ; 9.2 months later, MRI revealed local recurrence in the surgical cavity and a disseminated lesion in the upper thoracic spinal cord (Fig. 1G). Although BEV and palliative focal irradiation for the spinal cord lesion (20 Gy/5 Fr) were prescribed, the patient died 15.3 months after surgery.

### Histopathological characteristics of the tumors

**Fig. 2:**
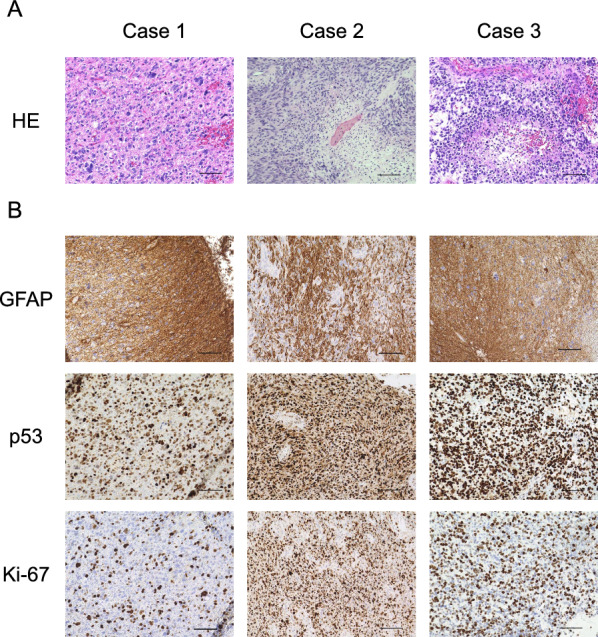
HE and IHC of the three tumors. **A** HE staining findings in Case 1 (left), Case 2 (middle), and Case 3 (right). Scale bar indicates 100 μm. **B** IHC using anti-GFAP antibody (top), anti-p53 antibody (middle), and anti-Ki-67 antibody (bottom) in Cases 1 (left), 2 (middle), and 3 (right). Scale bar indicates 100 μm

In all cases, the proliferation of atypical glial cells and mitosis were observed by hematoxylin and eosin (HE) staining of the tumor specimens (Fig. 2). Palisading necrosis and microvascular proliferation were observed in Cases 2 and 3, respectively. Tumor cells were positive for glial fibrillary acidic protein (GFAP) and p53 in all cases. Cases 2 and 3 showed especially strong p53 expression. The mean Ki-67 labelling index was 47% (range: 30–60). In Case 1, the pathological diagnosis was high-grade glioma based on the findings of atypical cells, mitosis, and a high Ki-67 labelling index (30%). In Cases 2 and 3, the pathological diagnosis was glioblastoma combined with atypical cells, mitosis, palisading necrosis, and microvascular proliferation.

### Molecular characteristics of the tumors

**Fig. 3:**
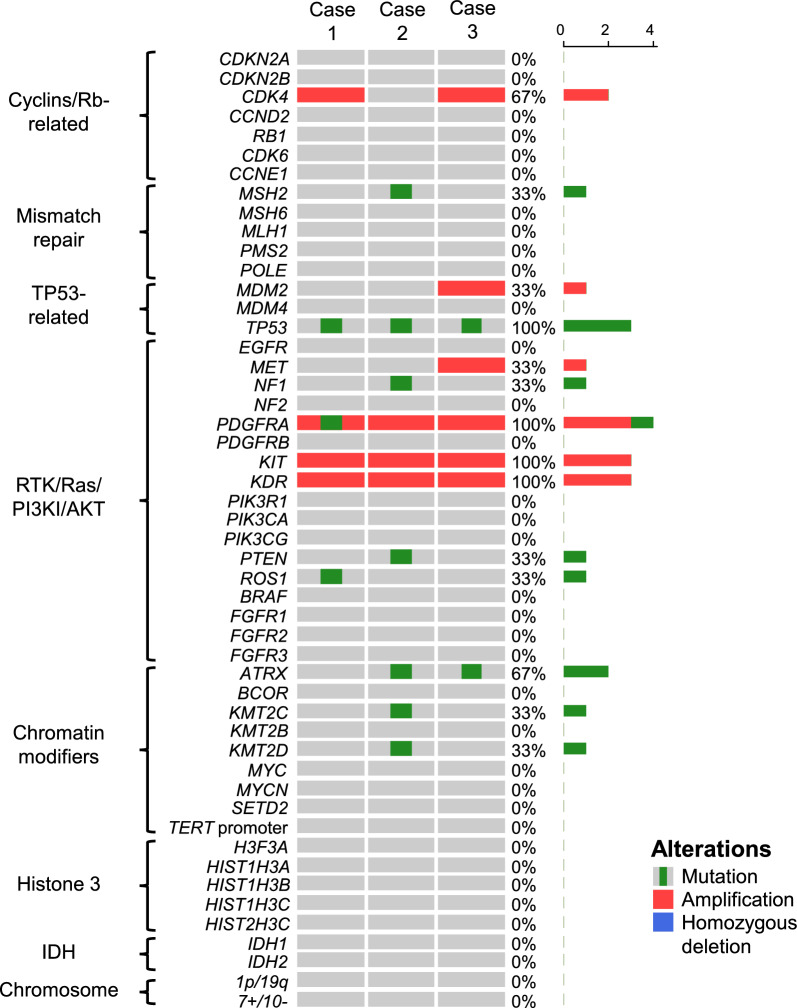
Gene mutations, copy number alterations, and chromosomal alterations in the three cases. WES data and methylation classifier data were used for mutation analysis and CNV analysis, respectively. Gene mutations (green box), focal gene amplification (red square), and homozygous deletion (blue square) of cyclins/Rb-related genes, mismatch repair genes, TP53-related genes, RTK/Ras/PI3K/AKT genes, chromatin modifiers, histone H3, IDH, and chromosomal alterations (1p/19q codeletion and chromosome 7 gain and 10 loss) in Case 1 (left), Case 2 (middle), and Case 3 (right). Right bar graph indicates the number of cases exhibiting alterations

Mutation analysis using WES data and CNV analysis using methylation classifier data from tumor tissues revealed no mutations in *IDH1*, *IDH2*, *BRAF,* or histone H3 genes (*H3F3A*, *HIST1H3A*, *HIST1H3B*, *HIST1H3C*, and *HIST2H3C*) in all tumors. *TP53* mutation and *PDGFRA* amplification were detected in all the samples (Fig. 3). However, *EGFR* amplification, 7 +/10− and *TERT* promoter mutation were not detected in any of tumors. *CDKN2A/B* homozygous deletion was also not detected in any of the tumors (Supplementary Fig. 1). Amplifications of *KIT* and *KDR* (*VEGFR2*) were detected in all tissues. Both genes are located on chromosome 4q12 along with *PDGFRA*. Case 1 harbored *PDGFRA* mutation and *CDK4* amplification. Case 2 harbored mutations in *MSH2*, *NF1, PTEN* and *ATRX* and *MET* amplification. Case 3 harbored *ATRX* mutation and amplifications of *CDK4*, *MDM2* and *MET * (Fig. 3 and Supplementary Table S1).

### TP53 mutation in the germline and tumors

**Fig. 4:**
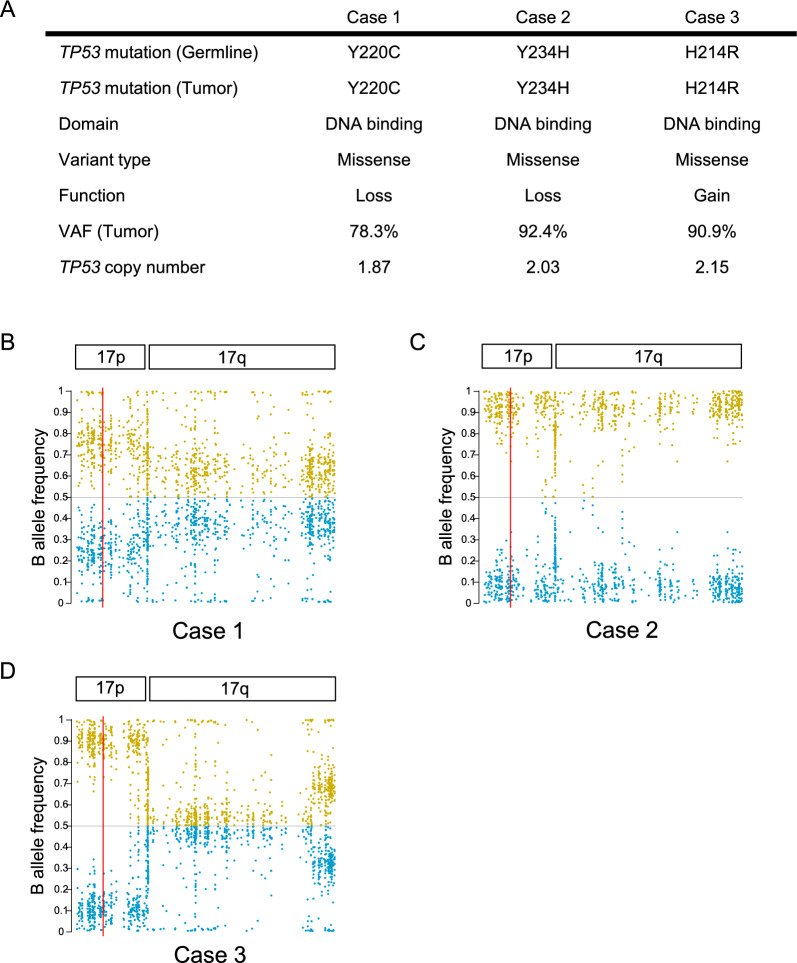
Status of *TP53* gene and chromosome 17 in the three cases. **A** Summary of *TP53* mutation patterns (germline and tumor), VAF (variant allele frequency) and copy numbers of *TP53* in Case 1 (left), Case 2 (middle), and Case 3 (right). **B**–**D** Dot plots indicating B allele frequency (BAF) of common SNPs on chromosome 17p and 17q in Case 1 (**B**), Case 2 (**C**), and Case 3 (**D**). Higher and lower mismatch ratios of the SNPs are shown as yellow and blue dots, respectively. A red line indicates *TP53* gene. All figures were obtained using WES data

All patients harbored heterozygous germline mutations of *TP53*. In all cases, mutation spots of *TP53* in the tumor-derived DNA were consistent with those of germline mutations (Fig. 4A). All mutations were missense mutations in the DNA binding domain of *TP53*. In all cases, tumor tissues showed high variant allele frequencies (VAFs) of *TP53* (range: 78.3–92.4%), while copy number alteration of *TP53* gene in tumor-derived DNA was not detected. Analysis of the BAF of SNPs revealed an allelic imbalance on chromosome 17 (Case 2) or 17p (Cases 1 and 3). These data indicated that the high VAF of *TP53* mutation is induced not by acquisition of a novel somatic *TP53* mutation in another allele that maintained heterozygosity, but by UPD on chromosome 17 or 17p, inducing loss of heterozygosity (Fig. 4B–D).

### DNA methylation profiling of the tumors

**Fig. 5:**
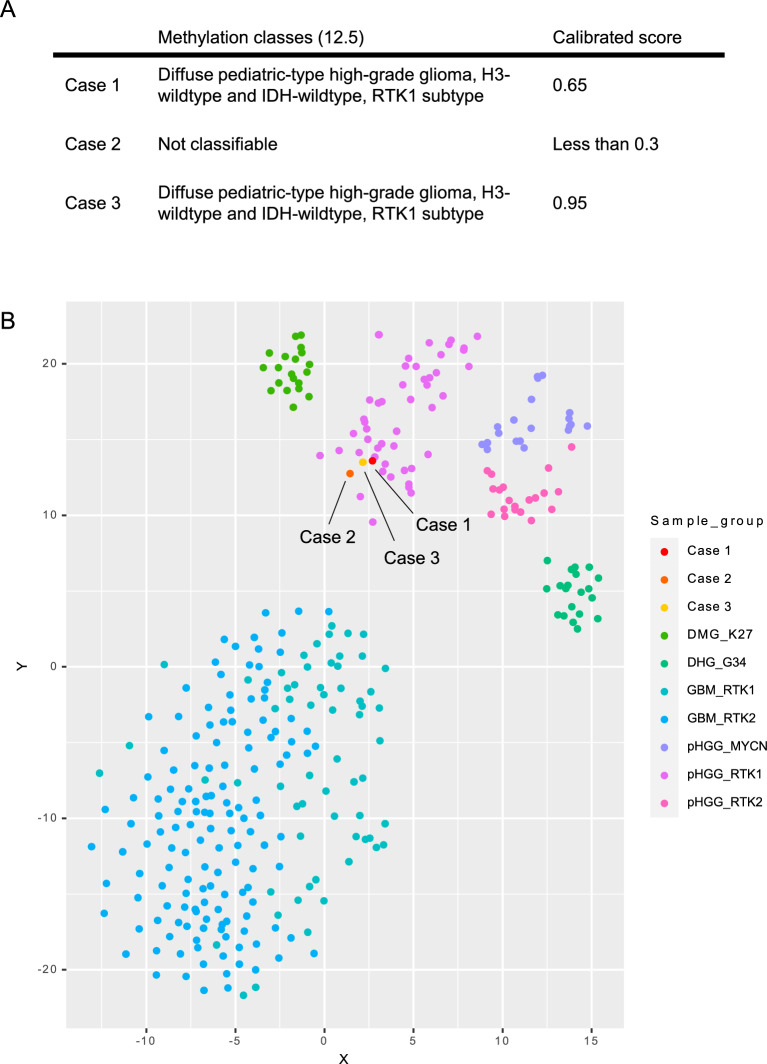
DNA methylation status of the three cases. **A** Table indicating methylation classes and calibrated scores obtained at the DKFZ classifier of Case 1, 2 and 3. **B** t-SNE plot indicating DNA methylation status of diffuse midline glioma, H3 K27-altered (DMG_K27; n = 20), diffuse hemispheric glioma, H3 G34-mutant (DHG_G34; n = 20), glioblastoma, IDH-wildtype (GBM_RTK1; n = 62, GBM_RTK2; n = 143) and pediatric-type high-grade diffuse gliomas (pHGGs), IDH-wildtype and H3 wildtype (pHGG_MYCN; n = 20 pHGG_RTK1; n = 20 and pHGG_RTK2; n = 20) including Case 1 (red), Case 2 (orange), and Case 3 (yellow)

DNA methylation-based classification using the DKFZ classifier (www.molecularneuropathology.org) revealed that Case 3 was matched to pHGG H3-/IDH-wt, RTK1 subtype with a high calibrated score (0.95). Case 1 did not match, but presented the highest calibrated score (0.65) for pHGG H3-/IDH-wt, RTK1 subtype. Case 2 was unclassifiable, with a low calibrated score (< 0.3), possibly because of the low quality of the DNA extracted from the FFPE sample (Fig. 5A). However, t-SNE analysis revealed that all cases clustered within pHGG H3-/IDH-wt, RTK1 subtype (Fig. 5B).

## Discussion

In this study, we investigated the clinical course and molecular features of high-grade gliomas that developed in three adult patients with LFS. All patients showed an aggressive clinical course despite multidisciplinary treatment. The location and radiographic features varied among the cases. In Cases 1 and 3, MRI revealed infratentorial lesions without contrast enhancement, which is not typical of adult-type glioblastoma, IDH-wildtype. In contrast, all tumors possessed typical histopathological features of high-grade gliomas, and two tumors did those of glioblastoma such as mitosis, palisading necrosis, and microvascular proliferation. WES of DNA derived from tumor tissues revealed no mutations in the *IDH* or histone H3 genes. Intriguingly, WES data also revealed no typical molecular features of glioblastoma, IDH-wildtype (*TERT*p mutation, *EGFR* amplification, and 7 +/10−) in all tumor tissues, whereas all tumor tissues harbored *TP53* mutation and *PDGFRA* amplification. Additionally, all tumor tissues clustered within pHGG H3-/IDH-wt RTK1 subtype in t-SNE analysis based on DNA methylation status, although Cases 1 and 2 did not exhibit significantly high calibrated scores for pHGG H3-/IDH-wt, RTK1 subtype using DKFZ classification system. Lower tumor cellularity is a major cause of lower calibrated score in the DNA methylation-based classification [18]. However, tumor cellularity of our cases was higher than 75% in all tissues, implying the presence of other reasons for the unmatched scores of Cases 1 and 2. Capper et al. reported that 12% of analyzed tumors could not be classified by DNA methylation profiling and this subset was highly enriched for unusual syndrome-associated tumors [14]. Other studies have also reported that a significant proportion (6–17%) of tumors, including pediatric or adolescent CNS tumors, could not be assigned to a classifier diagnosis [19–21]. These publications suggest that some cases of syndrome-associated tumors or pediatric-type tumors may not be accurately classifiable by DNA methylation profiling, although the entity of pHGG H3-/IDH-wt was not included in version 11.4 of the classifier. Since the introduction of the entity pHGG H3-/IDH-wt, Drexler et al. reported that 33.3% of cases that could not be classified by version 11.4 of the classifier, were not classified even by version 12.8, although only a small number of cases were classified as pHGG H3-/IDH-wt using version 12.8, including the entity of pHGG H3-/IDH-wt. They also reported that the t-SNE plot is also quite effective for the diagnosis of these unclassifiable tumors, beyond the matching score alone [22]. In addition, Guerrini-Rousseau et al. reported that tumor tissues from pediatric patients with LFS did not match the methylation classification but clustered in pHGG MYCN subtype using t-SNE analysis, as we did [23]. These findings suggest that for diagnosis based on the DNA methylation status of tumors arising in the context of a germline disorder, such as LFS, not only the DKFZ classification system but also t-SNE plot analysis is useful.

pHGG H3-/IDH-wt is a newly defined tumor type in the WHO CNS 5. pHGG H3-/IDH-wt comprises three subtypes (MYCN, RTK1, and RTK2). The RTK1 subtype has an intermediate prognosis (median OS, 21 months). The RTK1 subgroup is characterized by ahigh frequency of *PDGFRA* amplification. In The Cancer Genome Atlas (TCGA) database [24], among 362 adult GBM cases that were defined using the 4th edition of WHO classification whose gene mutations and copy numbers were available, only 14 cases (3.9%) harbored *TP53* mutation and *PDGFRA* amplification. These data might also support our findings that gliomas developing in adult patients with LFS have molecular profiles not like those of glioblastoma, IDH-wildtype but rather like those of pHGG H3-/IDH-wt.

There are a few other studies reporting that gliomas developed in adult patients with LFS. Tian et al. reported six IDH-mutant glioma cases and 13 IDH-wildtype glioma cases that developed in adult patients with LFS in a Chinese cohort, including three cases with *EGFR* mutation or amplification, and six cases with *PTEN* mutation, implicating glioblastoma, IDH-wildtype [25]. Among the other adult IDH-wildtype cases, no tumors harbored *PDGFRA* amplification. Two other studies have reported IDH-mutant astrocytoma cases in adult patients with LFS [7, 8]. Wu et al. reported a case of glioma in an adult patient with LFS, who possessed wild-type *IDH*, *H3F3A, TERT* and *EGFR*, and mutant *NF1* and *PDGFRB*, suggesting the potential of pHGG H3-/IDH-wt [26].

We also compared the *TP53* mutation status of tumor tissues with that of germline cells in each case. Mutation spots, copy number, and BAF of chromosome 17 suggested that UPD is associated with the development of gliomas in our cases. Recently, a high rate of copy number gains in *TP53* mutations was detected in tumors of patients with LFS [27]. This study revealed that the copy number gain of *TP53* variants occurs many years before tumor diagnosis, followed by accumulation of additional driver mutations that result in tumor development. Some studies have reported *MYCN* amplification in gliomas harboring wild-type *IDH* arising in pediatric LFS cases [8, 9, 23]. Our cases suggest that the copy number gain of mutant *TP53* obtained by UPD and *PDGFRA* amplification may play pivotal roles in the development of gliomas in adult patients with LFS.

In general, many mutant *TP53* proteins exhibit a dominant-negative effect on wild-type *TP53*, mostly by forming mixed tetramers with diminished DNA binding and transactivation activities [2, 28]. The mutation spots of *TP53* in our cases were not as frequent in LFS or brain tumors. The variants in Cases 1 (Y220C) and 2 (Y234H) have been reported to be associated with reduced transcriptional activity in the IARC TP53 database (http://p53.iarc.fr). Although the variant in Case 3 (H214R) has also been associated with reduced transcriptional activity, an increased ability to transactivate *GADD45* has been reported in a subset of breast cancer with this mutation [29]. However, no study has revealed an association between this mutation and gliomas. Our cases suggest that homozygous alteration of *TP53* by UPD might play a pivotal role in the oncogenic effects of some types of *TP53* variants, especially in the setting of LFS.

There is very little literature on pHGG H3-/IDH-wt because this is a rare tumor type. Additionally, it is quite difficult to distinguish this tumor subtype from glioblastoma, IDH-wildtype by histopathological and DNA mutation analyses. DNA methylation analysis is a unique and powerful tool for distinguishing this subtype from glioblastoma, IDH-wildtype. Although tumor predisposition syndromes are associated with pediatric-type cancers, the pathophysiology of adult-onset cancers in these patients is poorly understood. Our cases suggest that adult LFS patients also develop pediatric-type high-grade gliomas, and that *PDGFRA* amplification may have a high affinity for this type of glioma. Overall, this study revealed that atypical high-grade gliomas in adult patients with tumor predisposition syndromes, such as LFS, might be pediatric-type gliomas (Additional files 1 and 2).

## Conclusion

Adult high-grade gliomas, IDH-wildtype in the setting of LFS revealed DNA methylation profiles similar to those of pHGG H3-/IDH-wt. *PDGFRA* amplification and homozygous alterations in *TP53* may play pivotal roles in the development of this type of glioma in patients with LFS.

### Supplementary Information

**Additional file 1**. **Figure S1**: DNA copy numbers of three cases. Copy-number plots were generated using methylation classifier data. Dot plot indicates copy number of each location. Green or red dots indicate that the log2 copy number ratio is higher or lower than zero, respectively. The x-axis indicates location. The y-axis indicates the log2 copy number ratio. Dotted line indicates threshold of significant amplification (0.35) or deletion (−0.35).

**Additional file 2**. **Supplementary Table S1**: Log2 copy ratio in three cases.

## Data Availability

The datasets generated in this study are available based on request to corresponding author.

## Abbreviations

BAF: B allele frequency
BEV: Bevacizumab
CNS: Central nervous system
CNV: Copy number variant
FFPE: Formalin-fixed paraffin-embedded
GATK: Genome Analysis ToolKit
GFAP: Glial fibrillary acidic protein
HE: Hematoxylin and eosin
IHC: Immunohistochemistry
LFS: Li-Fraumeni syndrome
MAD: Median absolute deviation
MRI: Magnetic resonance imaging
OS: Overall survival
PFS: Progression-free survival
SNP: Single nucleotide polymorphism
TMZ: Temozolomide
t-SNE: T-distributed stochastic neighbor embedding
TTF: Tumor-treating field
UPD: Uniparental disomy
VAF: Variant allele frequency
WES: Whole exome sequencing
WHO: World Health Organization
