# Supplementary material for: Pediatric-type high-grade gliomas with PDGFRA amplification in adult patients with Li-Fraumeni syndrome: clinical and molecular characterization of three cases
Source: Acta Neuropathol Commun. 2024 Apr 11;12:57. doi: 10.1186/s40478-024-01762-7 (PMC11010357; doi:10.1186/s40478-024-01762-7)
Supplement: Supplementary file 1 — Additional file 1. Figure S1: DNA copy numbers of three cases. Copy-number plots were generated using methylation classifier data. Dot plot indicates copy number of each location. Green or red dots indicate that the log2 copy number ratio is higher or lower than zero, respectively. The x-axis indicates location. The y-axis indicates the log2 copy number ratio. Dotted line indicates threshold of significant amplification (0.35) or deletion (−0.35). [file 40478_2024_1762_MOESM1_ESM.pptx]

## Slide 1
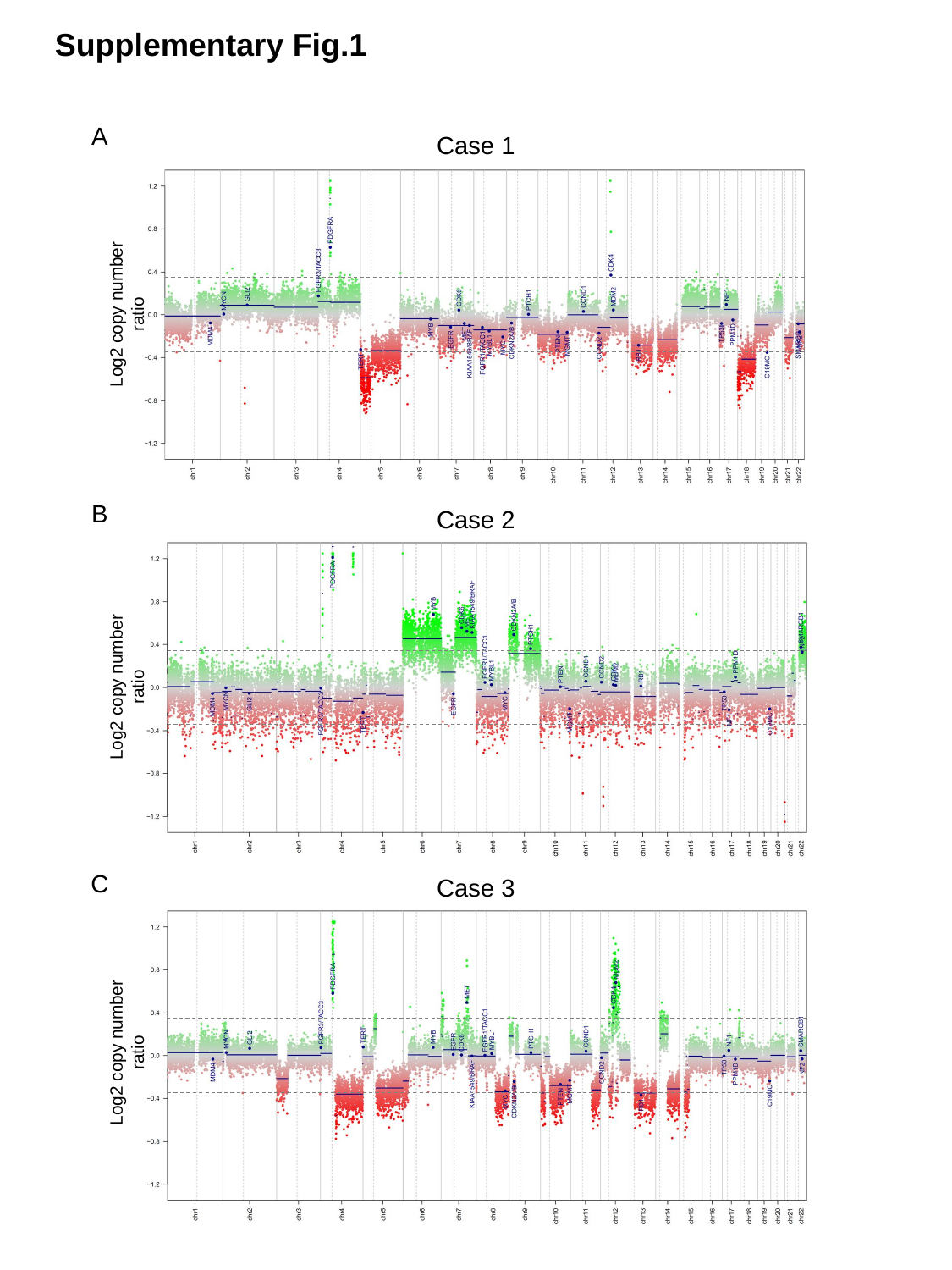

Supplementary Fig.1
A
Case 1
Log2 copy number ratio
B
Case 2
Log2 copy number ratio
C
Case 3
Log2 copy number ratio
